# Trait Anxiety Modulates Brain Activity during Performance of Verbal Fluency Tasks

**DOI:** 10.3389/fnbeh.2016.00010

**Published:** 2016-02-10

**Authors:** Barbara Gawda, Ewa Szepietowska

**Affiliations:** ^1^Department of Psychology of Emotion and Cognition, University of Maria Curie SklodowskaLublin, Poland; ^2^Department of Clinical Psychology and Neuropsychology, University of Maria Curie-SklodowskaLublin, Poland

**Keywords:** trait anxiety, verbal fluency, neuroimaging, executive functions

## Abstract

Trait anxiety is thought to be associated with pathological anxiety, and a risk factor for psychiatric disorders. The present study examines the brain mechanisms associated with trait anxiety during the performing of verbal fluency tasks. The aim is to show how trait anxiety modulates executive functions as measured by verbal fluency, and to explore the link between verbal fluency and anxiety due to the putative negative biases in high-anxious individuals. Seven tasks of verbal fluency were used: letter “k,” “f,” verbs, “animals,” “vehicles,” “joy,” and “fear.” The results of 35 subjects (whole sample), and 17 subjects (nine men, eight women) selected from the whole sample for the low/high-anxious groups on the basis of Trait Anxiety scores were analyzed. The subjects were healthy, Polish speaking, right-handed and aged from 20 to 35 years old. fMRI (whole-brain analysis with FWE corrections) was used to show the neural signals under active participation in verbal fluency tasks. The results confirm that trait anxiety slightly modulates neural activation during the performance of verbal fluency tasks, especially in the more difficult tasks. Significant differences were found in brain activation during the performance of more complex tasks between individuals with low anxiety and those with high anxiety. Greater activation in the right hemisphere, frontal gyri, and cerebellum was found in people with low anxiety. The results reflect better integration of cognitive and affective capacities in individuals with low anxiety.

## Introduction

Trait anxiety is a stable personality trait describing one's tendency to respond fearfully to a wide variety of stimuli (Spielberger et al., 1970). This is a general disposition to experiencing anxiety-relevant feelings or thoughts, or exhibiting anxiety-related behaviors (Spielberger, 1979). Highly trait-anxious people tend to perceive situations as more threatening, and they experience anxious states more frequently. They modify their perception of reality in such a way that they attribute a variety of stimuli with negative valence, and concentrate on these negatively perceived stimuli [the mechanism of attention inhibition (Öhman et al., 2000)]. Trait anxiety as a personality factor is associated with biological predispositions (e.g., Most et al., 2006; Öhman, 2008), pathological anxiety (Schmidt et al., 2008), and a risk factor for psychiatric disorders (Bienvenu et al., 2001). Anxiety and fear share many common physiological and cognitive properties, but may be distinguishable (Hartley and Phelps, 2012). Fear is a reaction to specific and short-term stimuli, while anxiety may be experienced in the absence of a direct threat, and lasts a longer period of time. Trait anxiety is a relatively consistent individual trait which is thought to be formed as a result of interaction between stress in early life and dispositional emotional arousal, which moderates the neuroplasticity of fear learning and memory (Kindt, 2014).

While the neurobiological bases of fear conditioning are well documented, the neural mechanisms of trait anxiety are not fully understood. However, these two topics are related because the development of trait anxiety is linked to the processes of facilitated fear conditioning and reduced fear extinction. People who are at risk of developing fear/anxiety disorders display impairments in extinction learning and reduced extinction memory (Kindt, 2014). The neural circuitry of fear conditioning has been extensively investigated in humans (Myers and Davis, 2002). Interestingly, the social fear learned through observation has similar neural mechanisms to those underlying classical fear conditioning (Olsson et al., 2007). The amygdala plays a central role in fear acquisition, storage, and expression. The amygdala is thought to be the site of association and storage of fear, with projections to the brainstem, the hypothalamus which mediates autonomic fear expression, and the ventral striatum which mediates coping with fear. In addition, the hippocampus and insula are important in the contextual modulation of fear. Then, the dorsal anterior cingulate cortex is thought to be involved in the modulation of fear acquisition. Fear circuitry also comprises some motor areas such as the primary motor cortex and dorsal basal ganglia (Butler et al., 2007). Cognitive-based fear engages motor control networks including the cortico-striato-thalamic loops. It reflects a state of motor readiness in response to danger (Butler et al., 2007). Control and/or extinction of fear is associated with the activity of the ventromedial prefrontal cortex (Peters et al., 2009; Sehlmeyer et al., 2009).

Although fear and anxiety can be distinguished, several theories propose that dysregulation of the neurocircuitry associated with fear conditioning are critically involved in the etiology and maintenance of anxiety (Mineka and Zinbarg, 2006). Trait anxiety is associated with increased amygdala activation and with elevated fear expression during fear acquisition (Lissek et al., 2005; Indovina et al., 2011). Anxiety also impairs extinction learning and emotional regulation of fear (Indovina et al., 2011; Sehlmeyer et al., 2011). In particular, this manifests in an inability to consistently inhibit fearful memories following extinction, and it results in a maladaptive expression of fear (Steinfurth et al., 2014). The role of the amygdala and the hippocampal formation in fear memory consolidation has been demonstrated (Albrecht et al., 2010), and amygdala activation is thought to be essential in selective attention to threat, as well as threat interpretation (Knight et al., 2009). In order to explain this process, researchers have considered the functional connectivity between the amygdala and prefrontal cortical regions (see Bishop, 2007). The medial prefrontal cortex, the dorsal ACC, and hippocampal areas comprise a part of the extinction circuitry (Hartley and Phelps, 2012). It is believed that these regions control the expression of fear by inhibiting amygdala activity. Anxious individuals show reduced response in both the rostral anterior cingulate region, implicated in detecting conflict from emotional stimuli, and in lateral prefrontal regions implicated in augmenting attentional control (Bremner et al., 2005; Rauch et al., 2005). Hyporesponsivity of the prefrontal regions and hyperresponsivity of the amygdala result in a disruption of the frontal-amygdala circuitry that, in turn, can influence attention, control, and interpretive mechanisms in highly anxious individuals (Morgan et al., 1993; Phelps et al., 2004). Trait anxiety associated with enhanced activation of the amygdala and decreased activity of dACC during late extinction learning was interpreted as delayed and reduced extinction of fear. This suggests that highly anxious subjects are not able to maintain inhibitory activation of dACC during the extinction process, this results in a failure to adapt to altering circumstances (Sehlmeyer et al., 2011).

Similar findings were shown for people who exhibited anxiety disorders: the hypo-activation of ACC during emotional processing (Etkin and Wager, 2007). Trait anxiety correlates inversely with the structural integrity of the vmPCF-amygdala pathway, suggesting an anatomical basis for heightened reactivity and impaired emotional regulation in anxiety (Kim and Wallen, 2009). The role of vmPFC in selective fear inhibition was shown; it inhibits fear response to one stimulus by facilitating the transference of this response on the currently predictive stimulus (Schiller et al., 2008). In addition, atrophy of the hippocampus in clinically anxious patients suggests that contextual modulation of fear may also be impaired. Thus, anxious individuals show an increased generalization of conditioned fear to similar stimuli (Lissek et al., 2010). A simple learned fear association may easily transfer to an overgeneralization of fear, however, trait anxiety is related to a generalization of fear responding only after an unpredictable aversive event (Kindt, 2014). Furthermore, a more sustained arousal and vigilance typical for anxious people is supported by the activation of the bed nucleus of the stria terminalis, which is a region in the ventral basal forebrain (Somerville et al., 2010). Moreover, the heightened perception of bodily sensation and interoception in anxious individuals appears to be associated with the role of altered insula which is thought to contribute to the maintenance of anxiety (Paulus and Stein, 2010).

The above mentioned results illustrate that the neural mechanisms of trait anxiety play an essential role for fear expression, sustained arousal, vigilance, heightened interoception, heightened reactivity, and impaired emotional regulation in anxious individuals. All these findings show that anxiety and trait anxiety may have an impact not only on emotional information processing, but also on cognitive processes. Recent neuroimaging studies suggest that the dysregulation of the fear conditioning circuitry and alterations in cognitive functioning in anxiety are based on the same neural mechanisms (Bishop, 2007). Two principal characteristics of information processing in anxious people were highlighted: a bias to pay attention to threat-related information, and a bias toward negative interpretation of ambiguous stimuli (Hartley and Phelps, 2012). Studies indicate that anxious people exhibit a tendency to facilitate the detection of threat-related stimuli, and a difficulty in disengaging attention from negative stimuli (Cisler and Kostner, 2010). In particular, when stimuli are more complex anxiety is related to a more negative interpretation (Bar-Haim et al., 2007). Likewise, differences in brain activity during positive vs. negative information processing were shown. During positive emotion processing, trait anxiety was found to modify neural activity in the right caudate head, and in the left superior temporal gyrus during the processing of negative emotion (Lemche et al., 2013). This biased attention in trait anxious people reflects increased amygdala activity to attended to threatening stimuli, as well as to unattended threat stimuli, and a decreased prefrontal activation under a condition of attention competition (Bishop, 2009). This altered threat sensitivity was documented by many studies (Bishop et al., 2004; Etkin et al., 2004; Haas et al., 2007). The threat-related biases, which are key-mechanisms of trait anxiety may develop as a result of abnormal safety learning in childhood, and they may be related to attention, appraisal, learning, memory, and threat sensitivity in adulthood. It is worth noting that these threat-biases are observed in anxious individuals at multiple levels of information processing (Britton et al., 2011).

Trait anxiety involves impaired attention and working memory. It involves perturbed attention allocation in the appraisal of potentially dangerous situations. This is supported by evidence of amygdala hyper-activation in anxiety disorders, and greater amygdala activation to negative stimuli, e.g., fearful faces (Britton et al., 2011). Associations between anxiety and memory were also documented. Appraisal processes are linked to cortical regions; activation of vmPFC during threat appraisal reflects the ability to discriminate between safety and threat; perturbations found in vmPFC activation during threat appraisal may reflect fear overgeneralization; finally a reduction in vmPFC activation is associated with poor long-term outcomes (Britton et al., 2011). Decreased positive amygdala-prefrontal functional connectivity was reported for young individuals with emotional dysregulation (Bertocci et al., 2014). Likewise, a large body of research suggests that anxiety may alter decision making, because uncertainty (associated with decision process) evokes threat-related information processing biases, and results in altered decision making (Hartley and Phelps, 2012). Anxious individuals are biased toward interpreting ambiguous contexts negatively (Grillon et al., 2004). Studies examining the neural substrates of processing ambiguity highlight the roles of the amygdala and the PFC; risk processing is more dependent on activity in the orbital prefrontal regions, whereas ambiguity processing recruits dlPFC (Krain et al., 2006). Ambiguity and risk processing are particularly aversive in anxious individuals. Studies also suggest that an increased level of anxiety is associated with greater loss aversion, because the above-mentioned pattern of brain activation seems to have a common underlying mechanism with an expression of fear and anxiety-related attentional biases (Hartley and Phelps, 2010, 2012). That is why anxiety increases the attention given to a negative choice option, negative interpretation of ambiguity, and the tendency to avoid potential negative outcomes which may inhibit flexibility of behavior (Hartley and Phelps, 2012).

Examining verbal fluency in anxious people may provide important information on their cognitive functioning; how they retrieve information, how they represent it, and organize it in memory. Verbal fluency tests are a measure of executive functions. The concept of executive functions refers to the top-down control of cognitive processes. The central executive component of the working memory model is characterized by an attentional control system (Larsson et al., 2007). Shimamura's (2000) dynamic filtering theory defines executive control as the monitoring, selection, and control of cognitive processes. Selection refers to the ability to direct attention toward a perceptual stimulus or a representation in memory. Maintenance refers to the ability to hold selected information active. Updating refers to the ability to modulate and reorganize information in working memory, and rerouting is associated with the ability to shift attention between different response sets. In a verbal fluency task, a participant is to generate words beginning with a specific letter (letter fluency), or belonging to a specific category (semantic fluency). Verbal fluency is dependent on both the ability to retrieve words from long-term storage and on executive functions. Shimamura (2002) pointed out that verbal fluency requires the ability to selectively focus attention on a semantic category, the ability to “on-line monitor” previously recalled words, and continuously update the words that have been used. Verbal fluency tests require an adequate mental set-shifting ability which guides the strategic search of words (Rende et al., 2002). Thus, the retrieval of semantic knowledge is dependent upon all domains of cognitive control because these domains are closely referring to attention and memory. Some data are particularly valuable, studies report close relationships between anxiety and cognitive functions, among them a relationship between anxiety and verbal information processing. For instance, verbal instruction may modify extinction processing which supports the idea that cognitive process is the primary mechanism of change during exposure therapy (Phelps et al., 2001). Hofmann (2008) reviewed empirical data and theoretical models suggesting that fear conditioning, fear extinction, and psychotherapy involve high-order cognitive processes. Thus, links between anxiety and cognitive processes are evident. First, because anxiety is conceptualized as a cognitive association of basic emotions, meanings, and responses (Barlow, 2002). Second, the neuroscience literature shows that cognitive process are critically important even in primitive forms of learning, thus it is not surprising that they are important in the acquisition and extinction of fear (Hofmann, 2008).

Due to verbal fluency's dependence upon executive control it seems to be reasonable to assume that the ability to retrieve semantic knowledge, as measured by verbal fluency, can be used to operationalize individual differences in executive control (Tabert et al., 2001). The results showed that the total number of words produced during verbal fluency tasks predicted the level of state anxiety, and it can be interpreted as support for a theoretical model of executive control capacity which may mediate emotional experience of state anxiety. Thus, an effective capacity to direct attention toward perceptual stimuli, or memory representations may be related to better retrieval or verbal coping strategies (Larsson et al., 2007). Links between executive functions and regulation of emotions are documented by neuroimaging studies. A high working memory capacity is related to an increased ability to resist putting attention on negative information. Thus, a high working capacity is characterized by a more effective attentional control (Derryberry and Reed, 2002). Personality traits such as trait anxiety may contribute to the ability to retrieve specific words. Rosen and Engle (1997) found relationships between verbal fluency and life-span working memory. There is also a study that suggests an association between personality traits and verbal fluency, i.e., Neuroticism was associated with lower scores in verbal fluency tasks (Sutin et al., 2011). Studies on verbal fluency in anxious and depressive people report that a high level of anxiety is associated with low verbal fluency scores in phonemic fluency tasks (Albus et al., 1998), both letter and semantic tasks (Beats et al., 1996), or only in semantic tasks (Fossati et al., 2003). Some cognitive impairments displayed by this group inhibit semantic strategies of retrieval and switching during verbal fluency performance (Atchley et al., 2003). Neuroimaging studies aim to show that dysfunctions of the prefrontal areas are thought to be involved in these low scores in verbal fluency tasks. Dysfunctions in activity of the prefrontal areas mirror the impairment of executive functions, and results in the use of non-effective retrieval strategies, and low switching capacities; this is reflected in low verbal fluency scores (Audenaert et al., 2002; Fossati et al., 2003). Anxiety has been found to be correlated with hypoactivation in the right prefrontal cortex in depressive patients, where verbal fluency tasks and neuroimaging techniques have been used (Liu et al., 2014).

Verbal fluency tasks may differ in their level of difficulty which may depend on the frequency of words (Ross, 2003; Ross et al., 2007). The general score in verbal fluency tasks is dependent on the frequency of the words as used in the general population; there are words of high frequency and they are generated quickly (in Polish the letter “k” is of high frequency, while “f” is of low frequency; Styczek, 1983). It means that there are less words in the Polish language starting with the letter “f,” and that is why this task would be more demanding than tasks letter the letter “k.” Then, tasks which include more typical words are easier than those including less typical words, the category “animals” is larger in terms of how frequently words are used, and more typical than the category “vehicles,” thus, it is easier to search the words from the lexicon of “animals” than from that of “vehicles” (Strauss et al., 1998). Furthermore, non-affective tasks are easier than affective tasks. Because language comprises more words naming animals, than words naming emotions, typically people generate more non-emotional words than emotional words (Tabert et al., 2001; Rossell, 2006). We introduce all types of tasks (letter, semantic, difficult, easier, emotional, and non-emotional) to analyze the potential effect of trait anxiety on modulation upon their performance, and the putative neural substrates of this modulation. To our knowledge, this type of study is the first.

We hypothesized that trait anxiety will differentiate performance of verbal fluency, thus we expect to see differences between low-anxious and high-anxious individuals in behavioral data. Horwitz and McCaffrey (2008) stated that verbal fluency performance in anxious people depends on the task's characteristics. Hence, we additionally expect that differences in the behavioral data will be more pronounced within high-anxious group, especially for difficult and emotional tasks (specifically differences between non-emotional and negative tasks are expected because high anxiety individuals exhibit negative attention biases). And then, we expect to see differences in brain activity during verbal fluency tasks between low-anxious and high–anxious groups, in particular while performing difficult, emotional tasks. Because of this the low-anxious people are thought to use more effective strategies to search, select, and retrieve words, we expect that they will activate more prefrontal regions across verbal fluency tasks, and they will present greater activation of these brain regions which are thought to be associated with the verbal fluency tasks' performance: the superior and the inferior prefrontal gyri, the temporal middle gyrus, the fusiform gyrus, the primary and secondary occipital cortex, the precuneus, and the superior parietal areas. Likewise, for emotional tasks, activation of some parts of the limbic areas is expected, such as the amygdala, hippocampus, or/and the cingulate cortex. Because of the general integrative role of the cerebellum in language, affective, and cognitive processing, the increased activation of the cerebellum is expected in low-anxious individuals.

## Materials and methods

### Participants

The results of 35 healthy, Polish-speaking, right-handed adults aged 20–35 (18 men and 17 women) were analyzed. Participants were paid for their participation. None of the subjects had a history of neurological or psychiatric disorders (each subject completed a questionnaire during a screening phase, with relevant information on neurological, psychiatric problems, and substance abuse). The selected participants were not addicted to drugs or alcohol (screening procedure). Handedness was verified using the Edinburg Inventory (Oldfield, 1971). The experimental protocol was approved by the Local Ethics Committee of the Department of Pedagogy and Psychology of the University of Maria Curie-Sklodowska. Participants had an average level of intelligence (*M* = 102, *SD* = 10) and no memory or attention impairments. Subscales from WAIS-R (Brzezinski et al., 2004; Vocabulary and Digit Span) were used to control these variables. The State Trait Anxiety Inventory was used to measure the level of trait anxiety. Two groups were identified on the basis of the trait anxiety score: a group with a high level of anxiety (*n* = 5; five men, 5 women) and another with low anxiety (*n* = 7; 3 men, 4 women). The high and low-anxious groups were selected on the basis of the normative data for the STAI; those participants who scored above 42 were classified as high-anxious, while those who scored below 32 were classified as low-anxious (Wrześniewski et al., 2002). These two groups representing the ends of the trait anxiety continuum were chosen to better illustrate the putative differences in brain activity during the performance of verbal fluency tasks. A categorical approach is helpful in communication and has a simplifying quality. Furthermore, clinical decisions regarding treatment are generally made with respect to a binary choice, as to whether or not a patient has a disorder. Whereas the low level of anxiety represents the low end of continuum which is adaptive, a high level of anxiety represents the second end of continuum where anxiety is non-adaptive (Endler and Kocovski, 2001).

In addition, trait anxiety as a dimensional variable was used in a simple regression analysis, as a predictor for neural activity.

### Procedure

**Figure 1:**
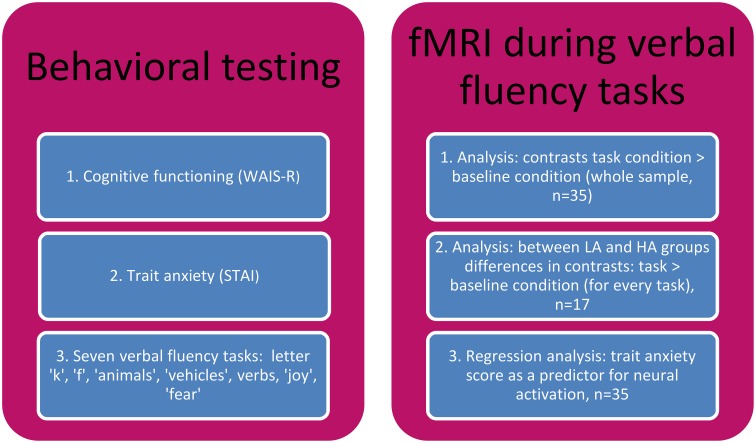
**The study outline**.

Verbal fluency tasks were administered to all the subjects before the scanning procedure took place. Then, STAI, and in addition a verbal IQ estimation test, Digit Span (WAIS-R) subscale, were administrated (study outline in the Figure 1).

#### Measures

##### WAIS-R

The Wechsler Adult Intelligence Scale-Revised is a general test of intelligence, based on 11 subtests divided into two parts: verbal and performance. Vocabulary and Digit Span scores were used in the screening procedure to qualify and compare working memory and verbal comprehension of the participants to select only those participants without any impairments.

##### STAI

The State Trait Anxiety Inventory. In this analysis only the Trait Anxiety score was considered. The Polish adaptation of the STAI consisted of 20 statements describing emotional conditions. The respondent is asked to rate the applicability of each statement to him/herself according to a 4-point frequency scale: 1-rarely, 2-sometimes, 3-often, 4-usually. The reliability and validity of the STAI are very good (Wrześniewski et al., 2002).

##### Verbal fluency tasks

The subjects' fluency was tested with seven tasks in the following order: letter “k,” letter “f,” “animals,” “vehicles,” verbs, “joy,” and “fear.” The subjects were asked to name as many words as possible in 1 min during the stage before scanning. All generated words were recorded by the experimenter, counted for every participant and for every task. All verbal fluency tasks were performed in the same order both inside and outside of the scanner (see Tables 1, 2).

**Table 1:** **The types of tasks**.

**Name of tasks (description)**	**Before the scan**	**During the scan (due to repetition of tasks, their equivalents were also used)**
Phonemic fluency: letter high frequency k (generating word s starting with a letter “k”)	X	letters: k, m, t
Phonemic fluency: letter low frequency f (generating word s starting with a letter “r”)	X	letters: f, g, n
Semantic fluency category living “animals” (generating nouns naming “animals”)	X	an imals, plants, birds
Semantic fluency category non-living “vehicles” (generating nouns naming “vehicles”)	X	vehicles, tools, furniture
Verbs (generating verbs)	X	X
Affective fluency positive “joy” (generating words representing the category “joy”)	X	joy, happiness, fun
Affective fluency negative “fear” (generating word s representing the category of “fear”)	X	fear, anxiety, fright

**Table 2:** **Descriptive statistics (*n* = 17)**.

**Variable**	GroupLA/^*^M(SD)	**Group HA *M*(*SD*)**	***t*_(1, 15)_**
Age	27.14 (5.26)	25.40 (4.30)	−0.45 ns
Education	14.70 (1.60)	14.20 (1.40)	0.15 ns
Vocabulary	32.00 (13.16)	25.00 (10.09)	0.16 ns
Digit span	4.57 (1.99)	3.30 (1.15)	1.71 ns
Positive verbal fluency: “joy”	11.14 (5.52)	10.70 (7.02)	0.14 ns
Negative verbal fluency: “fear”	10.85 (4.94)	10.40 (6.29)b	0.16 ns
Number of errors	1.08 (.91)	1.10 (0.92)	0.59 ns
Category “animals”	22.42 (7.13)	23.60 (6.18)	−0.36 ns
Category “vehicles”	16.00 (6.75)	13.90 (4.25)	0.79 ns
Verbs	18.57 (3.77)	17.70 (7.76)	0.27 ns
Letter high frequency	19.71 (3.63)	17.10 (3.84)	1.41 ns
Letter low frequency	16.14 (4.84)	12.09 (4.02)	1.92 ns
State Anxiety	29.85 (7.31)	35.10 (5.44)	1.06 ns
Trait Anxiety	30.00 (5.16)	49.10 (4.72)	7.90^***^

* *LA, low-anxious group; HA, high-anxious group; M, mean, SD, standard deviation, ns, not significant*,

*** *p < 0.001*.

#### fMRI procedure

##### Task, scanning procedure, and image acquisition

The examinations were performed in the magnetic resonance laboratory of the European Health Care Centre in Otwock (Poland). Each subject stayed in the scanner for approximately 30 min. Stimuli were presented to them in a blocked design with two alternating blocks: naming words silently (verbal fluency conditions) and to do nothing except look at a cross (baseline condition). During a sequence participants were asked to name as many items as possible related to the fluency categories which were equivalent to those used before the scanning procedure or to do nothing (baseline condition). Each sequence was preceded with instructions which were written in a textual format and shown on the screen. The LCD screen (NordicNeuroLab InroomViewingDevice) was used for visual presentation. There were seven identical functional sequences with different stimulation. Time of each sequence—3.18 min. There were repetitions of the following blocks in each sequence (each block lasted 6 vol.): 5 × (18-s display of gaze fixation point—cross, 18-s of active task) and 18-s of the cross at the end. There were seven different active tasks (see Table 1). The echo-planar images were acquired on a 3T Achieva Philips Medical Systems scanner using an 8-channel coil. The structural sequences (T1, T2) were assessed in order to exclude individuals with abnormal brain morphology; then high resolution T1 and SingleShot-EPI were used. The parameters of each sequence were as follows: T1 TFE high resolution sequence: TR = 7.51[ms], TE = 3.69[ms], FA = 8, FOV = 25.6 × 25.6 [cm], matrix = 256 × 256, slice thickness = 2[mm], gap = −1[mm], pixel bandwidth = 191 Hz/pix, number of slices = 181, TA = 3:18 min. A single-shot GE-EPI sequence was used for fMRI acquisition (FFE-EPI, TE = 30[ms], TR = 3000[ms], TA = 3:18 [min], slice thickness = 3[mm], gap = 0[mm], matrix = 96 × 96, FOV = 192 × 192 [mm], number of slices = 45, SENSE factor 1.8, dynamics = 66).

##### Image preprocessing

The fMRI data after transformation from DICOM to an analysis-compatible format were analyzed using the SPM12 package (Statistical Parametric Mapping). Data preprocessing comprised five consecutive steps: (1) a quality assurance procedure—checking images for artifacts and tSNR; (2) slice time correction (each slice was acquired in 67 ms in ascending order); (3) motion correction to eliminate motion artifacts—all images were realigned to the first image in the series, trials with motion above 2 mm were rejected (2/245 series), all realignment parameters were saved and used as the regressors within a GLM (general linear model) analysis, mean motion for all sequences was 0.47 mm, there were no differences between series in motion (mean motion for all 7 sequences: 0.52, 0.42, 0.41, 0.50, 0.44, 0.52, 0.45 mm), 7/245 sequences required a scrubbing procedure; (4) normalization of the brain images (anatomical T1-weighted images coregistrated with EPI—echo planar images) to MNI template (standard space suggested by the Montreal Neurological Institute, voxel size 2 × 2 × 2 mm) to enable between-group comparisons; and (5) smoothing filters (Gaussian kernel FWHM = 6 mm) were applied to decrease morphological differences between subjects. In the analysis the Automated Anatomical Labeling Atlas was used (Tzourio-Mazoyer et al., 2002).

##### fMRI data analysis

Two stages of analysis were performed: a single subject analysis (SSA) at a first level and a multi subject analysis (MSA) at a second level. Each EPI series had the same epoch-based paradigm. A GLM and a standard hemodynamic response function (HRF) were fitted to the data. The time-series for each voxel were high-pass filtered (1/128 Hz cutoff) to remove low-frequency noise and signal drift. To begin with, the first-level analyses were performed on individual subjects. The aim of this analysis was to show which regions of the brain were involved in the performance of the test, in other words to show whether there was a group effect of the performed test. In the first-level analysis one contrast was calculated “fluency task vs. cross.” This contrast was taken to the second-level analysis. A two sample *t*-test analysis, as well as a within-subject A two sample *t*-test was used to compare brain activation between two independent LA and HA groups (see Table 3), while a one sample Anova was used to compare brain activation across the whole sample (dependent variables were activations of brain regions). The main activations for contrasts tasks > baseline condition across the whole sample are presented in Table 4. The simple regression analyses were performed to better illustrate associations between trait anxiety and brain activation during the performance of difficult or/and easier tasks (results of regression in the text Section Results).

**Table 3:** **Brain regions which are more active for the contrasts of verbal fluency tasks > baseline condition, comparison between the LA and the HA groups (*n* = 17, two sample *t*- tests, *p* < 0.001, uncorr.)**.

**Clusters**			**MRI coordinates**			
**Verbs**	**Hemisphere**	**Active voxels (mm^3^)**	**x**	**y**	**z**	***t*-value**
Occipital Inferior gyrus	R	368	36	−82	−6	5.41
Cerebellum 8	R	328	20	−62	−56	4.74
Precuneus	R	144	4	−68	24	4.73
Inferior frontal gyrus BA 47	R	16	34	32	−20	4.08
Superior frontal gyrus	R	128	38	20	54	4.93
**ANIMALS**						
Fusiform gyrus	R	528	26	−66	−8	5.22
**VEHICLES**						
Cerebellum Crus 1	L	3240	−12	−90	−20	5.91
Temporal middle gyrus	R	344	52	−72	14	5.84
Cerebellum 6	R	936	22	−62	−16	5.75
Cerebellum Crus 1	R	808	44	−56	−38	5.65
Occipital middle gurus	R	384	34	−84	34	4.38
Fusiform gyrus	R	304	26	−62	−14	5.19
Cerebellum crus 2	L	448	−38	−68	−38	5.17
**POSITIVE FLUENCY JOY**						
Cerebellum 8	R	408	34	−68	−54	5.20
Cerebellum Crus1	L	560	−16	−86	−22	4.97
Occipital area BA 18	R	504	14	−94	−2	5.91
Occipital area BA 19	L	432	−30	−78	44	5.09
Superior parietal lobule	L	176	−56	−12	38	4.55
**NEGATIVE FLUENCY FEAR**						
Superior frontal gyrus	R	392	30	−76	−16	5.25
Cerebelum 6	R	400	34	−6	60	7.52
Fusiform gyrus	R	384	32	−76	−16	5.25
Thalamus	R	456	12	−8	12	5.59

**Table 4:** **Brain regions which are more active for the contrasts of fluency tasks > baseline condition (one sample analysis *t*-score, *n* = 35, *t*-threshold = 4.85, *p* < 0.05, FWE correction)**.

**Regions**			**MRI coordinates**			
**Verbs**	**Hemisphere**	**Active voxels (mm^3^)**	**x**	**y**	**z**	***t*-value**
Temporal superior gyrus	L	2168	−56	16	−8	14.82
Occipital inferior gyrus	L	2424	−28	−96	−8	13.01
Occipital middle gyrus	L	1720	−28	−96	−6	12.79
Cerebellum crus2	R	6376	28	−82	−48	12.25
Frontal superior gyrus	L	9688	−6	10	50	12.24
Frontal inferior gyrus	L	4744	−56	22	24	11.72
Cerebellum 8	R	3016	30	−68	−58	11.64
Occipital inferior gyrus	R	2424	38	−90	−12	10.98
Frontal superior gyrus	R	3880	2	6	64	11.12
Frontal inferior gyr. BA 47	R	2488	42	22	−6	10.20
**ANIMALS**						
Cerebellum crus1	R	10512	32	−68	−26	12.89
Cerebellum 6	R	3264	32	−68	−26	12.89
Cerebellum crus2	R	6720	8	−80	−28	12.37
Frontal superior gyrus	L	5512	−4	12	46	12.22
Cerebellum 8	R	2432	36	−66	−56	12.15
Anterior cingulate cortex	R	1592	−4	12	44	11.87
**LETTER K**						
Cerebellum 8	R	3624	34	−66	−58	14.10
Globus pallidus	L	1488	−18	6	6	13.95
Putamen	L	2384	−18	6	8	13.32
Frontal superior gyrus	L	5152	−4	10	48	12.49
Frontal inf. gyr. (pars oper.)	L	2304	−42	6	26	11.89
Precentral gyrus	L	4912	−44	6	24	11.86
Cerebellum 7b	R	1560	22	−78	−52	11.43
**LETTER F**						
Cerebellum 8	R	3624	28	−68	−58	12.68
Frontal superior gyrus	L	4656	−4	10	50	12.62
Frontal inf. gyr. (pars oper.)	L	2392	−42	6	26	11.85
Cerebellum 7b	R	1200	28	−74	−54	11.31
Temporal inferior gyrus	L	3248	−54	−52	−20	11.07
Putamen	L	2384	−22	8	6	10.95
Precentral gyrus	L	4888	−44	6	24	10.61
**VEHICLES**						
Temporal superior gyrus	L	1592	−56	16	−8	12.13
Cerebellum 6	R	2448	36	−64	−56	12.11
Precentral gyrus	L	5848	−46	6	32	11.11
Cerebellum Crus2	R	12376	38	−60	−32	11.09
Frontal inf. gyr. (pars tri.)	L	6696	−46	26	22	10.96
Frontal inf. gyr. (pars oper.)	L	3400	−40	6	28	10.53
Cerebellum Crus1	L	6912	−52	−58	−30	8.60
CerebellumCrus1	R	12376	38	−60	−32	11.09
Occipital middle area	R	496	26	−98	10	7.16
**POSITIVE FLUENCY JOY**						
Frontal superior gyrus	L	4840	−2	12	52	11.18
Occipital inferior gyrus	R	3848	38	−82	−12	11.15
Temporal superior gyrus	L	1232	−56	16	−6	11.14
Insula	L	1792	−28	20	4	10.22
Cerebellum 8	R	1920	34	−68	−54	10.08
Cerebellum Crus1	R	8000	38	−74	−26	9.95
Calcarine sulcus	R	1352	18	−96	2	9.89
Cerebellum Crus1	L	928	−30	−84	−18	7.84
Occipital lobe BA 19	L	2864	−28	−94	6	9.02
**NEGATIVE FLUENCY FEAR**						
Frontal superior gyrus	L	7024	−4	12	48	13.09
Temporal superior gyrus	L	1432	−54	16	−8	12.97
Cerebellum Crus1	R	10960	26	−76	−24	12.54
Occipital middle gyrus	R	1720	38	−92	4	12.12
Anterior cingulate cortex	L	1384	−4	14	44	11.30
Occipital middle gyrus	L	3624	−34	−94	4	11.06
Calcarine sulcus	R	1840	20	−102	4	11.05
Cerebellum 6	R	2136	28	−70	−26	10.85
Frontal superior gyrus	R	1848	2	10	54	9.05
Fusiform gyrus	R	192	30	−82	−6	7.38

## Results

### Behavioral data

There were no significant differences between the groups with low anxiety (LA) and high anxiety (HA) in terms of age, education, Vocabulary, Digit Span, number of words in positive verbal fluency, number of words in negative verbal fluency tasks, number of words in the categories of “animals,” “vehicles,” number of verbs, and number of words from the phonemic fluency (both low frequency and high frequency letters; see Table 2). The two groups differed significantly in trait anxiety, but not in state anxiety. There were also no significant sex differences in the abovementioned variables (*t* = 0.94, *p* = 0.33).

The within-group comparisons (a Wilcoxon test) for the HA group showed significant differences between scores in more difficult tasks and easier tasks (with the Bonferroni correction). Their scores were higher in easier tasks, such as: category “animals” as opposed to the harder category “vehicles” (*z* = −2.80, *p* < 0.001), letter “k” in contrast to the more difficult letter “f” (*z* = 2.60, *p* < 0.01), and verbs over category “animals” (*z* = 2.31, *p* < 0.01). These comparisons show that “vehicles,” verbs, and category letter “f” are more difficult tasks for the HA, whereas “animals” and category letter “k” are easier. The most difficult category seems to be that of “vehicles.” Similar comparisons for the LA group did not show significant differences between “vehicles,” “animals,” verbs, and letter categories (the letter “k” to the letter “f,” *z* = 1.40, ns.), “animals” to verbs (*z* = 1.44, ns.), and “vehicles” to verbs (*z* = −1.24, ns). The above results show that for the LA group there are no differences between the difficult and easier tasks, whereas these differences are found for the HA group. This supports the thesis that HA individuals differ in cognitive processing between more complex and less complex tasks.

The within-group comparisons between performance on emotional (“fear,” “joy”) and non-emotional tasks (non-emotional means the categories of letters “k” and “f”) were assessed separately within the LA and HA group. These comparisons showed that the LA group on average generated more words starting with the letter “k” than words in the category “fear” (*z* = −2.37, *p* < 0.01), and they generated more words starting with the letter “f” than in the category “fear” (*z* = −2.38, *p* < 0.01). The HA group generated more words starting with the letter “k” than in categories “joy” and “fear” (*z* = −2.37, *p* < 0.01, *z* = −2.45, *p* < 0.01, respectively). No significant differences were found for the comparisons between the number of words starting with the letter “f” and categories “joy,” “fear” within the HA group. The mean number of words for the HA group is presented in Table 2. The above findings show a typical tendency: that people generate more non-emotional than emotional words, and that HA individuals did not generate more negative nor positive words than non-emotional. This is not in line with data suggesting negative attention biases in anxious people.

### Neuroimaging data

Interestingly, no differences were found in all behavioral data (all tasks) between the LA and the HA groups, yet differences in brain activation during the verbal fluency tasks were identified. To compare neural correlates between verbal fluency tasks between the LA and HA groups, a two sample *t*-test was used.

**Figure 2:**
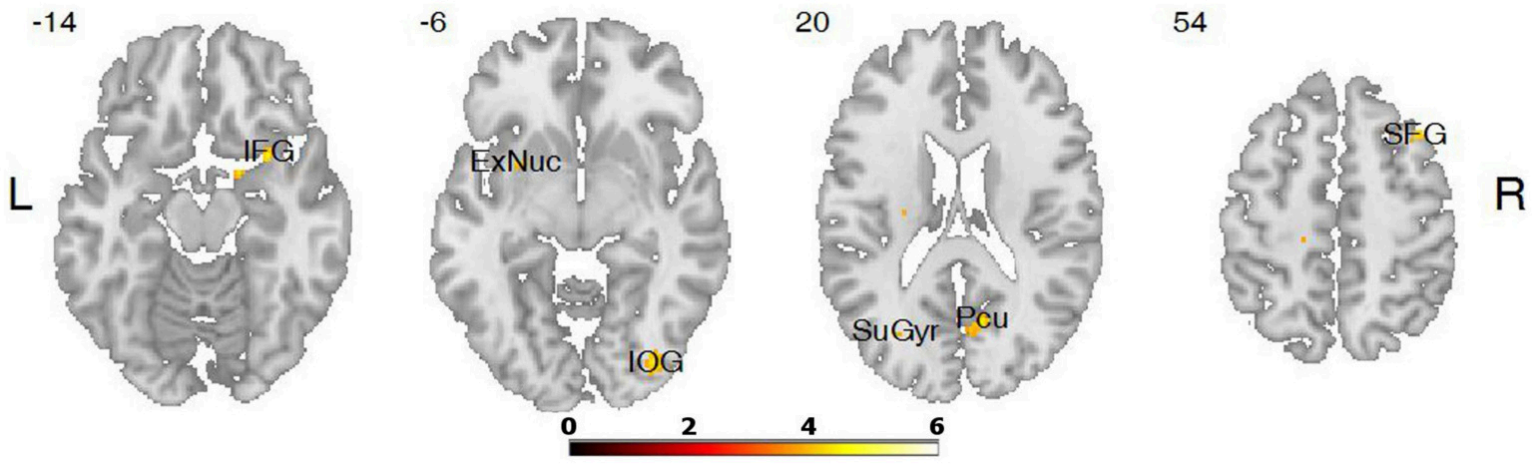
**Differences in brain activation between LA and HA groups for the contrasts verbs > baseline condition: in the right inferior frontal gyrus (IFG), right inferior occipital gyrus (IOG), right precuneus (Pcu), right superior frontal gyrus (SFG)**.

**Figure 3:**
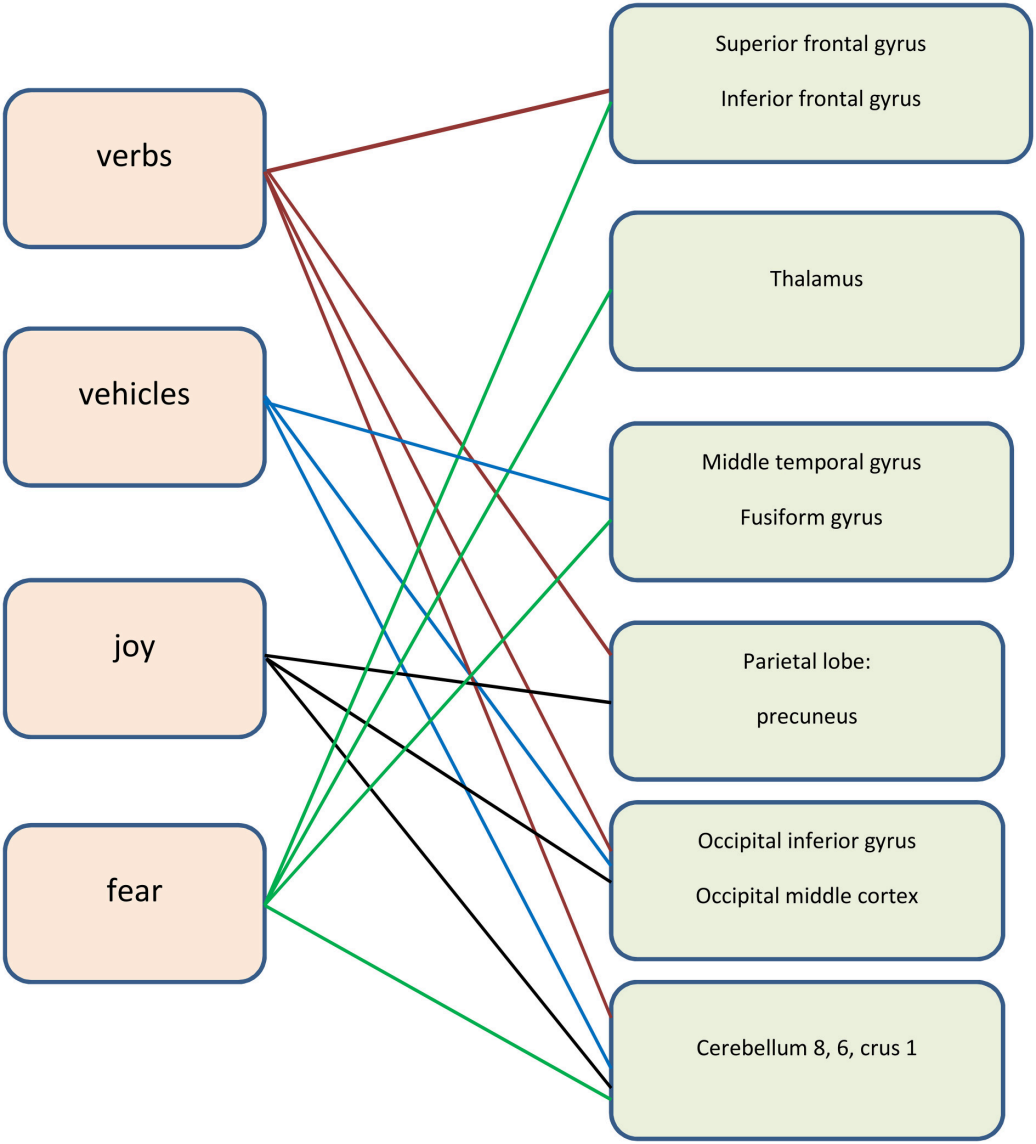
**The neural areas involved in the tasks (the right hemisphere)**.

**Figure 4:**
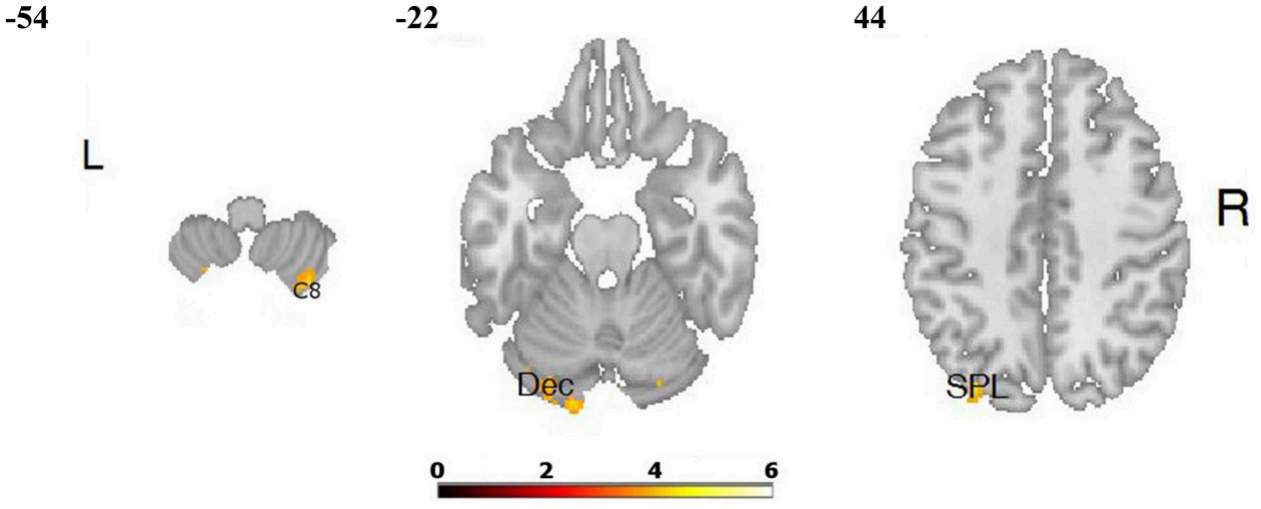
**Differences in brain activation between LA and HA groups for the contrasts “joy” > baseline condition: in the right cerebellum 8 (C 8), left cerebellum (declive—Dec), left superior parietal lobule (SPL)**.

The differences in activation during the **verbs** task were found in five clusters when the following thresholds were adopted: *p* = 0.001 (uncorrected), *t*-threshold = 3.73, cluster size threshold = 38, alphasim *p* < 0.05. In the case of the LA group, greater activation was found in the right occipital inferior gyrus, in the right cerebellum 8, in the right precuneus, in the right superior, and in the inferior frontal gyri (see Table 3, Figures 2, 3). A predominance of activation in the right hemispheric regions was observed in the LA group during this task, and similarly during all of the more difficult tasks. The illustration of these active regions in the right hemisphere is presented in Figure 4. In addition, a simple regression analysis across all participants revealed a significant weak negative correlation between trait anxiety and activation in the right occipital inferior gyrus (36, −82, −6), in the right cerebellum 8 (20, −62, −56), in the right precuneus (4, −68, 24), and in the right superior gyrus (38, 20, 54).

Another more difficult category is that of “**vehicles**,” where several differences in activation between the LA and HA groups for this task were found. Activation for “vehicles” comprises the right and left cerebellum, the right temporal gyrus, the right fusiform gyrus, and the right middle occipital gyrus. A simple regression analysis with trait anxiety as predictor showed that trait anxiety is negatively and strongly correlated with the activation in the cerebellum during the performance of task “vehicles.” A negative weak correlation between trait anxiety and activation in the temporal areas and activation in the occipital areas was found. It suggests that higher trait anxiety is associated with lower activation of the aforementioned brain regions.

Less demanding tasks such as that of “**animals**,” the **letter “k,”** and the **letter “f”** did not elicit differences in brain activity between the LA and HA groups as it was hypothesized. Brain activation during the “animals” task was greater in the LA group only in the right fusiform gyrus. Then, the high frequency letters task elicited no differences in activation between the LA and HA groups. Similarly, low frequency letter task caused no differences in activation between LA and HA. This was surprising.

**Figure 5:**
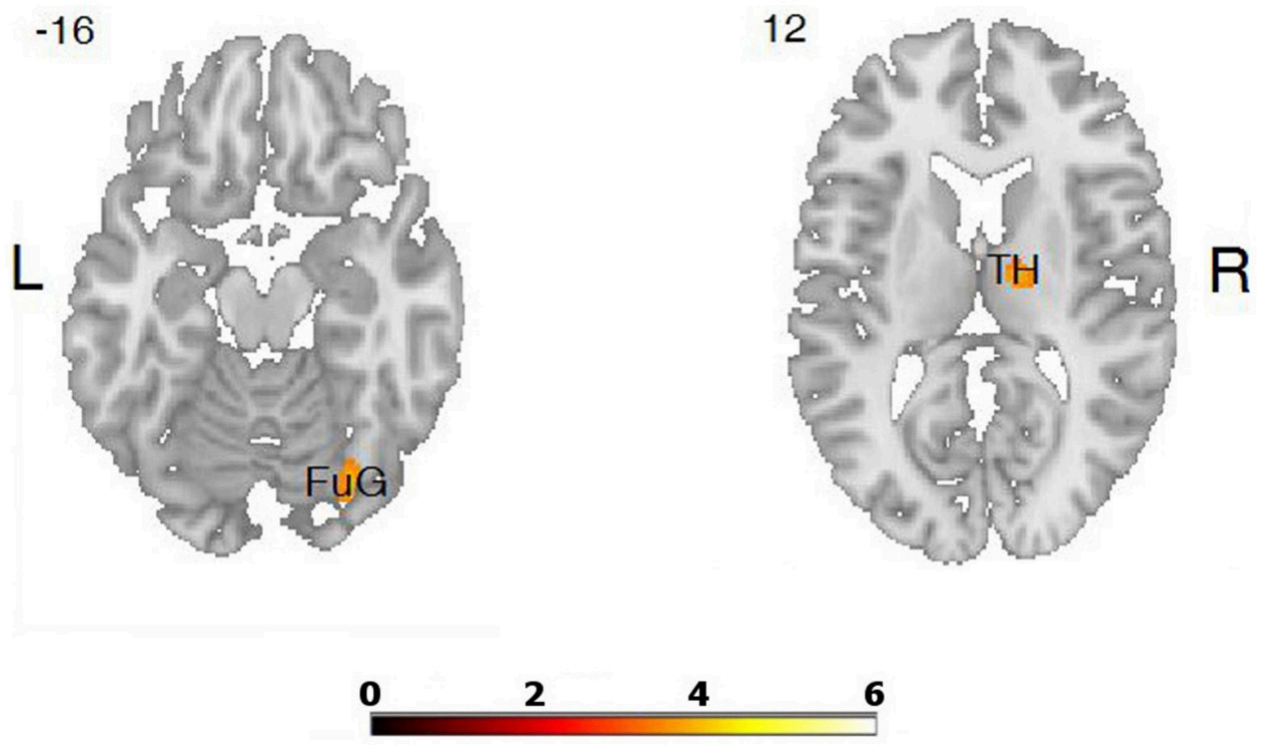
**Differences in brain activation between LA and HA groups for the contrasts “fear” > baseline condition: in the right thalamus (TH), right fusiform gyrus (FuG)**.

The comparisons of brain activity during the performance of the emotional tasks between the LA and HA groups showed some differences. In the case of the category “**joy**,” a predominance of activation in the right hemisphere was not observed, whereas it was in category “**fear**.” The performance of positive verbal fluency tasks in the LA group elicited higher activation in the right cerebellum 8, left cerebellum crus, the secondary visual cortex (the right occipital area BA 18, the left occipital area BA 19) and in the left parietal lobule. In sum, five clusters were found when the following thresholds were adopted: *p* = 0.001 (uncorrected), *t*-threshold = 3.73, cluster size threshold = 38, see Figure 4. Negative verbal fluency tasks in the case of LA individuals elicited greater activation in the right frontal superior gyrus, in the right fusiform gyrus, the right cerebellum, and the right thalamus. For category “fear” four clusters were found when the following thresholds were adopted: *p* = 0.001 (uncorrected), *t*-threshold = 3.73, cluster size threshold = 38, alphasim *p* < 0.05 (see Table 3, Figure 5). Additionally, a simple regression analysis was conducted with trait anxiety as a predictor. It showed strong negative correlations between trait anxiety and activation in two regions: cerebellum crus1 (−16, −86, −22) and the occipital area BA 18 (14, −94, −2). Other negative correlations for emotional verbal fluency categories were weak, but they consistently show that with higher trait anxiety the activation is lower in the same brain regions as it was shown by the *t*-test comparisons.

## Discussion

The purpose of this study was to describe whether trait anxiety modulates brain activity during verbal fluency task performance. The differences in neural activation between LA and HA individuals were found, while no differences in behavioral data between these groups were identified. It suggests that different neural mechanisms may be involved in retrieval processes, yielding similar behavioral effects (Bishop, 2009). LA and HA individuals may employ other neural strategies to achieve the same results: for LA individuals it is easier to be well concentrated, while for HA people is harder to achieve the same result, activating more inappropriate brain regions, possibly HA group execute tasks with greater effort. It is in line with findings that show that trait anxiety impairs processing efficiency more than performance effectiveness (Derakshan and Eysenck, 2009). Possibly it refers also to the first general finding which was not expected: the predominance of right hemispheric activation during almost all difficult tasks in the low-anxious subjects. It may be explained in terms of data highlighting the right hemisphere's associations with explicit memory retrieval (Gabrieli et al., 1998). Possibly low-anxious individuals use more explicit and more self-relevant strategies during the performance of verbal fluency tasks. The right prefrontal cortex is associated with self-relevance during subjective evaluation, people engage more episodic retrieval during tasks of subjective evaluation (Schmitz et al., 2004). Predominance of the right hemisphere in the majority of tasks may also be interpreted as the use of novel, non-typical strategies by low-anxious people (Garoff et al., 2005). It may refer to their use of more visual strategies during verbal fluency tasks performance (Goldberg et al., 2013).

The next general finding, which was hypothesized, suggests a relationship between the task difficulty and the differences in brain activity in the LA and HA individuals: the easier the tasks the lower the differences between the LA and HA groups. This suggests that easier and more familiar tasks, such as high frequency letters and “animals” did not elicit many differences in brain activity between the groups. On the other hand, more difficult tasks coincided with more differences in brain activity between LA and HA, i.e., harder tasks such as verbs, “vehicles,” “joy,” and “fear” elicited more differences between the neural mechanisms of the LA and HA groups. In addition, in the HA group, significant differences between scores in the more difficult tasks and easier tasks were found. It supports the thesis that HA individuals differ in information processing between more complex and less complex tasks, and these differences are reflected in neural mechanisms. It is in line with the former findings that anxiety has a negative effect on complex or difficult tasks (Mayer, 1977), and with more recent findings (Horwitz and McCaffrey, 2008; Hartley and Phelps, 2012). It shows that trait anxiety slightly modulates brain activity associated with cognitive processes such as executive functions. For instance, the ability to selectively focus attention on a semantic category, the capacity to monitor recalled words, and/or continuously update the words that have been used. A complex tasks may elicit an uncertainty in anxious people, and this cognitive state may evoke threat-related information processing biases, and results in altered information processing (Hartley and Phelps, 2012). Studies on ambiguity, loss aversion, and risk processing in anxious individuals support the present findings that increased anxiety is associated with involvement of the different cognitive and neural mechanisms in demanding and easier tasks. Association between the difficulty of tasks and trait anxiety may be explained in terms of the cognitive noise thesis; cognitive noise may interfere with working memory (Robinson and Tamir, 2005). Authors of this thesis state that trait anxiety elicits cognitive noise which reduces cognitive flexibility in anxious people. On the contrary LA individuals do not exhibit such problems, and this was reflected in our results as lack of differences between scores in difficult and easier tasks.

We did not confirm the differences in scores for the LA and HA groups in non-emotional and emotional tasks; the within-group comparisons did not show any difference, especially in the HA group where negative verbal fluency have higher scores. However, differences in neural activity between emotional and non-emotional tasks within the LA and HA group were found. Our results present greater activation of the prefrontal regions in LA than in HA individuals, as it was hypothesized (the frontal superior gyrus in the “fear” category, and increased activity the frontal superior gyrus and frontal inferior gyrus in verbs). This seems to be linked with better attentional capacities and an unimpaired monitoring process in low-anxious individuals. Trait anxiety reduces such functions by impairing attention and task-switching capacity (Eysenck et al., 2007). Our findings correspond with data presented by low-anxious subjects as found with increased activation in the fronto-parietal networks, while highly anxious individuals showed a particular pattern of increased functioning of the cingulo-opercular and ventral attention (Sylvester et al., 2012). Attentional control theory presents the idea that anxious individuals show weaker, and insufficient or stronger (supposedly compensatory) neural activation in brain regions supporting attention (Basten et al., 2012). Greater activation of the frontal regions during verbal fluency tasks in the LA group, and lower activation of these regions in the HA group may reflect not-impaired attention, better working memory and information processing in LA individuals. Low anxiety does not involve perturbed attention allocation in appraisal (Britton et al., 2011), altering in decision, or lack of flexibility (Hartley and Phelps, 2012). Low anxiety is associated with not-impaired executive functions which refer to top-down control of cognitive processes. Increased activity of the frontal regions in group LA suggests that they engage in more effective monitoring, selection, and control of cognitive process (Shimamura, 2002). To perform verbal fluency tasks effectively the ability to selectively focus attention on a semantic category and the ability to “on-line monitor” previously recalled words and continuously update the words are all required. These abilities refer to the directed attentional system which is responsible for top-down control of attention and partly to a stimulus–driven attentional system (because instruction during study changes). These two systems are regulated by the different brain regions; top-down control of attention involves prefrontal regions of the brain, whereas a stimulus–driven attentional system engages the temporo-parietal and ventral frontal cortex (Corbetta and Shulman, 2002). These two systems interact in their functioning (Pashler et al., 2001). Effective attentional capacities require reciprocal influences of each system on the other. Anxiety may impair the balance between these two attentional systems (Corbetta and Shulman, 2002). The possibility of good balance between these systems in LA individuals in our studies is reflected in the increased activation of the frontal regions in the LA group and less differences in neural activity between difficult and easier tasks in this group.

Another noteworthy element is the fact that low-anxious individuals seem to use more adequate strategies, and they will activate brain regions which are thought to correspond with these strategies, as it was hypothesized. For example, the prefrontal cortex is thought to be involved in autobiographical memories, it modulates the amygdala-hippocampus network in the initiating, searching, and monitoring of memory (Dolcos et al., 2012). Medial and orbital prefrontal cortex activity is more associated with emotional retrieval (Markowitsch et al., 2003). These parts of the brain are connected with the thalamus to regulate memory and emotions (Barbas, 2000). These parts of the brain were more activated by the low-anxious people during the performance of verbal fluency tasks; verbs and emotional tasks. Furthermore, retrieval of words is linked to the posterior areas such as the parietal and occipital regions associated with the visual-spatial processing of information, including the processing of emotional information (Dolcos et al., 2012). And these regions were activated by the low-anxious people. Moreover, the brain areas typically involved in verbal fluency tasks, such as the middle temporal gyrus (which is thought to be responsible for semantic processing; Birn et al., 2010) and the fusiform gyrus also involved in semantic processing (Ardila et al., 2006; Noppeney, 2008; Pulvermüller, 2013; Ralph, 2014) were more active in the low-anxious individuals during verbal fluency performance. All these differences in activation between the LA and HA groups, as it was expected, support the claim that low trait anxiety enables the use of more adequate neural strategies of retrieval. The possibility that neural activation differences for LA and HA subjects could be due to differences in task-related effort inside the scanner is unlikely because the comparisons between the task condition and baseline condition (which are the indirect measure of effort) show a lot of significant differences. These comparisons show that subjects executed tasks with adequate effort, as we see in the Table 3, all activated brain regions are those which are typically activated during verbal fluency performance.

We did not confirm the increased activation of the limbic areas during emotional verbal fluency tasks, with one exception. Only the right thalamus was more activated during the “fear” tasks in LA individuals. The activation of the thalamus in the “fear” tasks may be interpreted in terms of Rolls' concept of the implicit-explicit emotional language. He stated that implicit emotional language is associated with activation of the thalamus, premotor, cingulate, and striatum, while explicit emotional language involves more temporal and frontal areas (Rolls, 1999). Our results show that the negative verbal fluency associated with greater activation of the thalamus, may be thought of as more implicit than the positive category. Higher activation of the amygdala and hippocampus was not found, which may be explained in the light of the recent findings. Involvement of the amygdala in emotional encoding is well-documented, however, its involvement in the retrieval of emotional memories has been difficult to demonstrate (Dolcos et al., 2012). This is because activation of the amygdala also depends on the intensity of emotional retrieval, and retrieved information such as those in our verbal fluency tasks may not be excessively charged. Higher intensity of emotional memories is associated with activation in both the amygdala and hippocampus (Botzung et al., 2010).

As it was hypothesized, our findings also show an important role of the cerebellum during retrieval in LA people. Its activation was greater especially in the difficult tasks such as “vehicles” and “joy.” The cerebellum, through the connections with the prefrontal, parietal, temporal, and cingulate cortex, regulates many functions such as episodic memory, imagination, executive functions, as well as language processing (Habas et al., 2009; Stoodley et al., 2012). Our finding is in line with other evidence which shows cerebellar activation in relation to language, attention, affection, emotion, and mental imagery, and that the cerebellum is able to integrate multiple internal representations with external stimuli and self-generated responses. The cerebellar modulation permits the production of harmonious motor, cognitive, and affective behaviors. This is possible because more than half of the cerebellar cortex is interconnected with association zones of the cerebral cortex (Schmahmann and Sherman, 1998). The role of the cerebellum is well-documented, for instance, patients with cerebellar cognitive affective syndrome (which is linked to cerebellar lesions) display deficits in cognitive functioning, spatial cognition, visual-spatial memory, language, personality, and behavioral reactions, as well as affective disturbances ranging from emotional blunting and depression to disinhibition (Mariën et al., 2009). The integrative role of the cerebellum is highlighted by hypothesis of the functional cerebellar-encephalic pathways (Mariën et al., 2009). This concept holds that the cerebellum facilitates an automatic modulation of behavior, and the behavior being modulated is determined by the specificity of anatomic subcircuits within the cerebro-cerebellar system. The posterior cerebellum is involved in cognitive processes when the vermis is thought to be the limbic cerebellum. The cortico-ponto-cerebellar pathways are linked to the adjustment of emotional and cognitive process to situational context (Parvizi et al., 2001). Thus, damage to the cerebellar components of the neural circuits subserving sensorimotor, cognitive, or emotional processing disrupts the universal cerebellar transforming functions and causes the accompanying cognitive-affective deficits (Schmahmann, 2004). This shows that the cerebellum is involved in the emotional congruency, emotional regulation, cognitive flexibility, and working capacities (Annoni et al., 2003).

Greater activation of the cerebellum found in low-anxious people during the difficult verbal fluency tasks may reflect better integration of cognitive and affective capacities in low-anxious individuals, compared to the high-anxious people. In general, individuals with a high level of trait anxiety exhibit a lower level of integration of emotional and cognitive capacities (Öhman, 2008).

## Limitations

The first limitation of this study was the small sample size. Second, potential factors influencing the sex differences in emotionality. We included nearly an identical number of women and men in the HA and LA groups, not find significant sex differences in cognition (by WAIS-R), and sex differences in trait/state anxiety. The results should be however interpreted with caution because of potential not-included factors influencing brain activity, such as menstrual cycle phase which was not taken into account, and might influence female brain activity (Comasco and Sundström-Poromaa, 2015).

## Conclusion

The above findings confirm that trait anxiety slightly modulates brain activity during the performance of verbal fluency tasks. The acquired evidence shows that trait anxiety has an impact on attention, working memory, and strategies for retrieving information from memory. This impact reflects the differences in the neural mechanisms employed by low-anxious and high-anxious people, and may be observed especially during the performance of the more difficult tasks. Greater activation of the prefrontal regions, the cerebellum and the typical brain areas associated with the kind of verbal fluency task in low-anxious people reflects their better ability to selective focus attention on a semantic category, ability to perform “on-line” monitoring of recalled words, updating and switching capacities. In sum, low-anxious individuals seem to activate more adequate neural strategies of retrieval. Anxiety impairs processing efficiency more than performance effectiveness, thus anxious people may have similar behavioral results but employing information processing strategies different from non-anxious people (Derakshan and Eysenck, 2009). It may suggest that they exhibit the easier use of novel, non-typical strategies, and that they employ sensory-visual strategies more effectively, even in self-referential aspects, in comparison to highly anxious people (Northoff et al., 2006). The presented results highlight the better integration of cognitive and affective capacities in low-anxious individuals.

Our findings increase understanding trait anxiety as incorporated not only in mental organization, but also in neural representation, and as affecting cognitive functioning. They establish verbal fluency tests (with fMRI) as a useful tool in the assessment of brain mechanisms in anxious people, and/or anxiety disorders.

## Author contributions

All authors listed, have made substantial, direct and intellectual contribution to the work, and approved it for publication.

### Conflict of interest statement

The authors declare that the research was conducted in the absence of any commercial or financial relationships that could be construed as a potential conflict of interest.
